# Platelet‐Rich Plasma Injection Combined With Q‐Switched Ruby Laser in the Treatment of Periorbital Hyperpigmentation

**DOI:** 10.1111/jocd.16598

**Published:** 2024-09-25

**Authors:** Yankun Lu, Danyi Huang, Ting Liu, Li Yang, Yiming Lin, Xiaomin Fang, Han Ma

**Affiliations:** ^1^ Fifth Affiliated Hospital of Sun Yat‐Sen University Zhuhai China

**Keywords:** periorbital hyperpigmentation, pigmentary disorder, platelet‐rich plasma, post‐inflammatory hyperpigmentation, Q‐switched ruby laser

## Abstract

**Background:**

Periorbital hyperpigmentation is a prevalent skin condition that represents a large quantity of cases seen in cosmetic dermatology. Patients tend to be left with pigmentation after Q‐switched ruby laser treatment, especially the perioribital area.

**Aims:**

The study is conducted to compare the effect of PRP injection combined with laser therapy versus laser alone for periorbital hyperpigmentation treatment.

**Patients/Methods:**

In this single‐center, case–control study, 30 patients with periorbital hyperpigmentation were allocated to receive PRP injection injection after Q‐switched ruby laser or Q‐switched ruby laser only, followed by a 12‐week and 24‐week follow‐up visit. Visual analogue scale, Sadick tear trough rating scale, and patients' self‐evaluation were used to evaluate the therapeutic effect.

**Results:**

The combined group achieved a better improvement in long‐term effect and had effect on facial rejuvenation. Patient satisfaction was higher in the combined group. Erythema and mild pain were the most common adverse reactions of both groups.

**Conclusions:**

Combining with PRP injection can improve the therapeutic effect of Q‐switched ruby laser in treating periorbital hyperpigmentation and lessen the risk of post‐inflammatory hyperpigmentation, indicating a new option for POH treatment.

## 1 Introduction

Periorbital hyperpigmentation (POH) is a common skin disorder that accounts for a significant proportion of patients in cosmetic dermatology. It is multifactorial in etiology, such as inadequate sleep, allergic contact dermatitis, familial and periorbital edema [1]. POH can be classified into four types: pigmented, vascular, structural, and mixed type based on the clinical appearance [2]. In recent years, laser interventions have been one of the most published of any treatment modalities for POH, and it is a widely accepted method for the treatment of pigmentation. The most commonly used lasers are neodymium‐doped yttrium aluminum garnet (Nd:YAG) laser, Q‐switched ruby (QSR) lasers, erbium yttrium scandium gallium garnet (Er:YSGG) laser, and cutaneous CO_2_ lasers. Among these, QSR laser is the representative equipment which can break the subepidermal melanosomes into pieces and release melanin particles into the cytoplasm, and then cleared by macrophages [3]. And the 694‐nm wavelength of the QSRL is more strongly absorbed and more selective for melanin. However, while laser treatment is effective in treating hyperpigmention, they can also worsen symptoms and contribute to post‐inflammatory hyperpigmentation (PIH) [4]. It was shown in a study that Nd:YAG laser caused PIH in 36.4% of patients, and 29.4% patients developed PIH after QSRL therapy [5, 6]. Asian population seem to have a higher tendency toward PIH. Furthermore, PIH is more likely to be present in the periorbital area, as the superficial vasculature and thin skin overlying orbicularis oculi [7]. Some studies even suggesting that QSR laser is not recommended for dark‐skinned population [8]. Therefore, it is an urgent need to find out an optimized therapeutic regimen based on Q‐switched ruby laser treatment, which can achieve improvements for patients with dark circles and minimize the risk of PIH.

Platelet‐rich plasma (PRP) refers to a small amount of plasma containing a high concentration of platelets and growth factors. It was obtained from autologous blood by centrifugation, and the concentration of platelets in PRP is three to five times that of normal plasma [9]. It has been shown that PRP can improve skin damage caused by UV radiation and promote repair after laser surgery [10]. Also, Mehryan et al. [11] performed a study which had proved PRP injections itself has the effect of improving POH and found improvement in both color homogeneity and melanin content. Therefore, in this study we tried to use PRP injection after QSRL therapy, hoping to achieve the purpose of maximize the efficacy of combined treatment, improve PIH after QSRL treatment, and provide a new method for clinical treatment of periorbital hyperpigmentation.

## 2 Materials and Methods

Thirty patients age ranged from 24 to 33 years, Fitzpatrick skin type III–IV, diagnosed with periorbital hyperpigmentation (pigmentary subtype predominant) participated in this prospective, randomized case‐control study. The sample size was determined based on the available literature. Recruitment started on October 1, 2021 and follow‐up was completed on December 31, 2021 at the Department of Dermatology, Fifth Affiliated Hospital of Sun Yat‐Sen University. Patient informed consent was obtained from all participants. Participants meeting all the following criteria were eligible for the study: (1) pigmentary dark eye circles or mixed dark eye circles with predominantly pigmented subtype; (2) Fitzpatrick skin type II–IV; and (3) age above 18 years old. Participants were excluded from the study if they (1) have any signs of eye infection or inflammation, (2) have history of photosensitive drugs or periocular injections or surgery in the past 12 months, (3) have a platelet count < 150 × 10^9^/L, (4) could not avoid sun exposure within 4 weeks before and after treatment, and (5) are pregnant or lactating.

Platelet‐rich plasma was prepared in the Department of clinical laboratory in Fifth Affiliated Hospital of Sun Yat‐Sen University. Before preparation, the operating room was disinfected with a UV lamp for 60 min, the biosafety cabinet for 30 min, and the preparation materials for 30 min. A sterile tube containing EDTA‐K2 anticoagulant with 8 mL whole blood was extracted from each subject in combined group. A 2‐mL sample of EDTA‐K2 anticoagulated blood was used for complete blood cell analysis to determine the baseline platelet concentration. The remaining 6 mL underwent the first centrifugation at 500* g* for 10 min. Then the upper layer of platelet‐rich plasma was aspirated into a 15 mL centrifuge tube, labeled as “plasma,” the platelet concentration in the plasma was measured using an automated blood cell analyzer and recorded. The plasma was centrifuged at 2200* g* for 16 min again, after centrifugation, the upper layer was poor platelet plasma (PPP), and the platelet at the lower layer was mixed with PPP to achieve a final PLT concentration of approximately (1000 ± 20%) × 10^9^/L, labeled as PRP. The platelet concentration in the PRP was measured and recorded using an automated blood cell analyzer. The final volume of PRP was calculated using the formula: PRP final volume = plasma PLT count (10^9^/L) × plasma volume/1000 × 10^9^/L. One milliliter PRP product was mixed with 0.1 mL calcium gluconate (containing 100 U/mL of thrombin), incubated in a sealed condition at 37°C in a water bath with shaking for 40 min, and then centrifuged at 2200* g* for 20 min. The supernatant was then transferred to a new centrifuge tube and labeled as “activated PRP product.” The remaining PRP product was stored at −80°C for sample preservation. The reference value of platelet concentration in whole blood in normal population is 125–350, and the three to five times the basal platelet concentration is within a fluctuation range of 450–1750. In order to improve the comparability of clinical trials, we used a fixed value as the quality control target, the target value is 1000 ± 10%, an exceeding 1000 ± 20% was regarded as out of control. The processed specimens were stored in a 2°C–6°C refrigerator for 7 days.

Twenty patients were randomized to receive PRP injection after QSR laser (combined group) and 10 patients received QSR laser only (control group). A random draw generated by computer was used to determine which group the patient belonged to and was enrolled by a separated researcher. The treatment was given once a month for a total of three sessions and the follow‐up visit was set after the last treatment (12 weeks) and 3 months after the last treatment (24 weeks). Lidocaine cream was applied around the eyes for 40 min for routine disinfection. QSR laser (Ruby Laser system SINON; Alma Lasers) was used to scan the inferior orbit three to four times. Set the parameter mode: QSW, spot: Pixel 5 × 5, set power: 22–24 mJ/Pixel (adjusted according to patient's skin type, higher power for lighter skin), frequency: 1.0 Hz. In combined group, PRP was applied to the dermis of the inferior orbit using a 30 G needle at an approximate depth of 1.5–2.0 mm, the injection sites were approximately 1 cm apart, and the injection volume was approximately 0.1 mL per site, resulting in a total injection volume of approximately 1–1.5 mL each subject. Ice pack was applied to each patient for 30–60 min after operation to prevent pain and skin burn after laser treatment. To reduce the risk of postoperative hyperpigmentation and infection, all patients were instructed to protect themselves from the sun strictly and the local wound should not be stained with water for 3 days. All patients were photographed by Visia skin analysis system (Mindscan, CBS Taiwan) before treatment (baseline, Week 0) and two additional follow‐up visits at 12 and 24 weeks after treatment.

**FIGURE 1:**
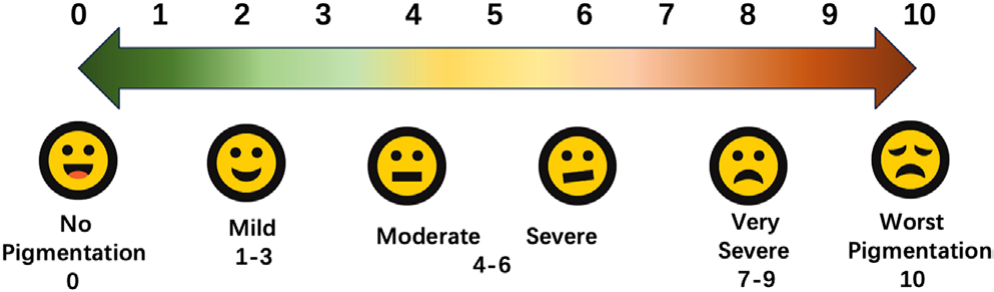
Visual analogue scale.

For clinical analysis, the primary outcome is visual analogue scale (VAS) for hyperpigmentation evaluation (Figure 1). Treatment success was defined as a significant difference between before and after treatment. The secondary outcome include Sadick tear trough rating scale (TTRS), which include depth of the trough, suborbital hyperpigmentation, nasal fat pads and pockets, and lower eyelid skin rhytidosis [12]. Patients self‐evaluation was also recorded at each follow‐up visits. Photographs of the patients taken by Visia skin analysis system and the two rating scales were evaluated by two experienced dermatologists. This study was approved by the bioethics committee of the Fifth Affiliated Hospital of Sun Yat‐Sen University and registered by Chinese Clinical Trail Registry.

Database management and statistical analysis was performed with SPSS statistics version 22.0. Paired *t*‐test was used to analyze the VAS score and TTRS of patients at baseline and each follow‐up visit. Chi‐squared test was used to compare the satisfaction of patients between two groups. Any value of *p* < 0.05 was considered statistically significant.

## 3 Results

A total of 30 patients were included in the study. The average age was 28.9 years (median 29, SD 2.66). Nine patients had Fitzpatrick phototype III and twenty‐one had phototype IV. The average duration of POH was 2.48 years (median 3.0 ± 0.75). There were no significant differences in baseline between the two groups. Detailed information is shown in Table 1.

**TABLE 1:** Clinical characteristics of the patients.

Variables	*n* = 30 (%)
Age	28.9 ± 2.66
Fitzpatrick phototype	
III	9 (30)
IV	21 (70)
Duration of POH	
≤ 1 year	4 (13.3)
> 1, ≤ 2 years	8 (26.7)
> 2 years	18 (60)
Family history of POH	3 (10)
History of insomnia	2 (6.7)
Other atopic disease	4 (13.3)
Melasma	0 (0)

### 3.1 Primary Outcome

**FIGURE 2:**
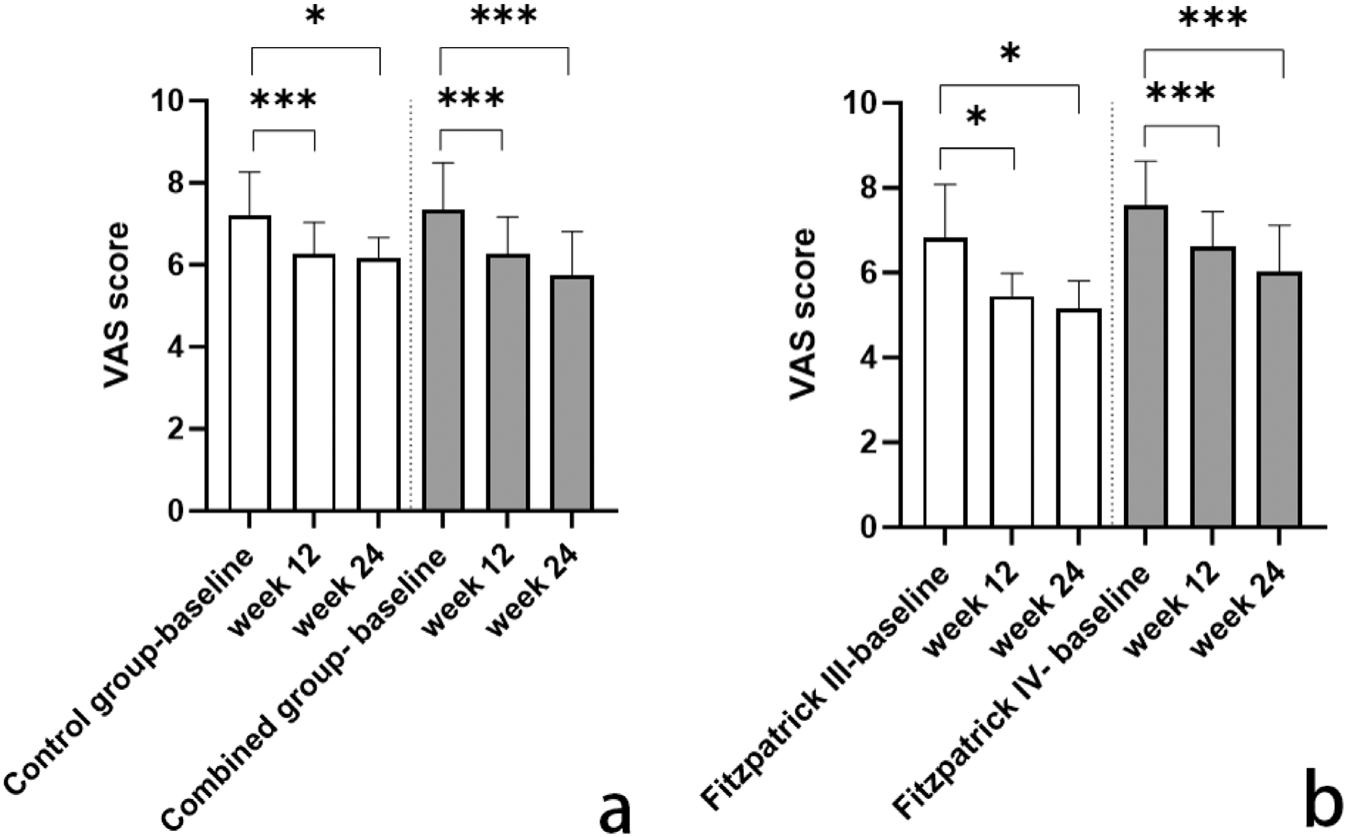
Differences in visual analogue scale score in control group and combined group (a) different outcomes in Fitzpatrick types III and IV (b) *p < 0.05, ***p < 0.001.

The VAS of the combined group was 7.36 ± 1.13 at baseline, and the score after 12 and 24 weeks was 6.26 ± 0.92 and 5.76 ± 1.05. For the control group, VAS was 7.21 ± 1.05 at baseline, and 6.25 ± 0.79 and 6.17 ± 0.50 at 12 and 24 weeks (Figure 2). There was no significant difference in the baseline level between combined group and control group. The difference between baseline level and after treatment in both groups were statistically significant, indicated that both treatment are effective. The difference observed in the combined group was more significant compared to the control group. For long‐term evaluation, the difference in combined group between 12 and 24 weeks were statistically significant, but there was no significant improvement in the control group, indicating that PRP injection may have a long‐term improvement against POH and PIH. We also explored the differences of VAS between Fitzpatrick type III and IV and the results showed that both groups were effective but there was no difference between the two groups (Figure 2).

### 3.2 Secondary Outcome

**FIGURE 3:**
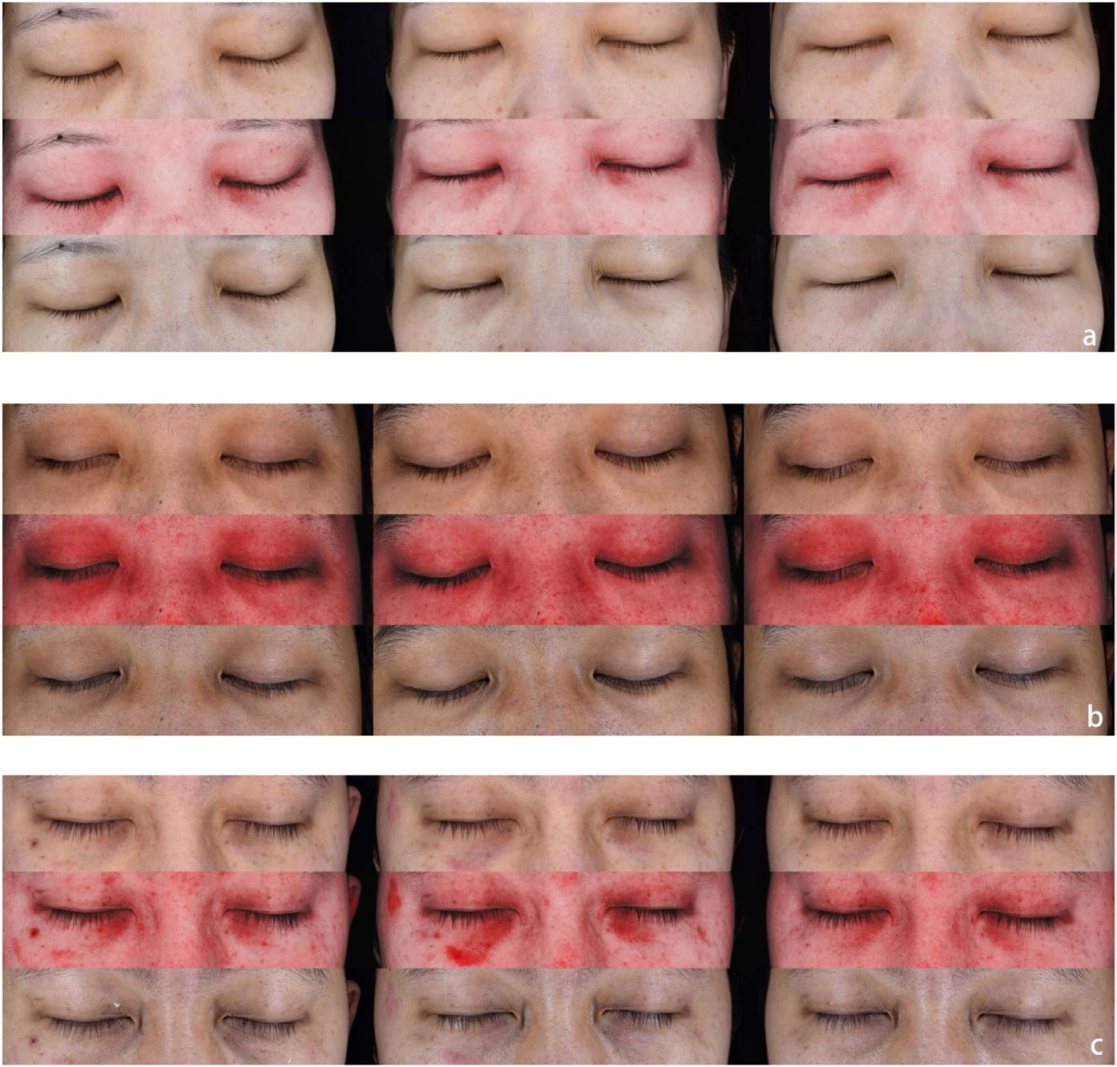
Two patients in combined group with Fitzpatrick phototype III and IV (a, b). The patient in figure a was scaled as 7.7 at baseline and 5.7 and 5.3 at 12 and 24 weeks, the patient in (b) was scaled as 8.7 at baseline and 6.7 and 6.4 at 12 and 24 weeks. One patient in control group with Fitzpatrick phototype IV (c) was scaled as 7.7 at baseline and 7.3 and 7.3 at 12 and 24 weeks.

At the final visit, the patient self‐evaluation of 17 patients were “very satisfied,” two patients were “moderately satisfied,” and one patient was “slightly satisfied” in combined group, and in control group the self‐evaluation of four patients were “very satisfied” and five were “moderately satisfied,” and one “dissatisfied.” The patient self‐evaluations of the combined group were higher than those of the control group, and the difference was statistically significant (*χ* ^2^ = 14.089, *p* = 0.002). The Sadick TTRS of combined group at baseline was 12.90 ± 2.47, and 11.08 ± 2.21 and 10.38 ± 2.44 at 12 and 24 weeks. In control group, the baseline score was 11.6 ± 1.84 at 12 weeks and 11.70 ± 1.81 and 11.15 ± 1.65 at 24 weeks. In the combined group, there was a significant improvement in before and after treatment, indicated that PRP injection after laser therapy could also improve facial rejuvenation. In contrast, the control group showed no treatment effects, and the score did not indicate significant difference. Also, some other values base on Sadick TTRS such as melanin deposition, wrinkles, tear trough, and local inflammation were recorded using Visia skin analysis system, and showed a definite improvement in combined group. From the top to the bottom line, the figure from left to right shows the PL spectra, red spectra and RGB spectra of Visia skin analysis system at baseline, 12 and 24 weeks (Figure 3).

### 3.3 Adverse Effects

The common adverse effect after operation in both group was erythema and mild pain (30 of 30, 100%), which could be relieved immediately after ice compression. No severe adverse reactions such as pigmentation, infection, and scar were observed. There was no regional complication reported in any application of PRP in combined group patients.

## 4 Discussion

There is a variety of therapeutic alternatives for POH. According to the results of previous studies, topical preparations containing LMW‐HS, hydroquinone, vitamin K, vitamin C, retinol, and chemical peelers can be used as the choice [13]. Lasers also have good therapeutic effects on vascular and pigmented POH. Since the ruby laser used firstly to treat human skin in the early 1960s, the selective photothermolysis theory led to the development of variety lasers, and become the standard method for many skin pigment changes. Q‐switched ruby laser can selectively target melanosomes with minimal damage to other tissue and have no associated with scarring, atrophy or cutaneous depression, and it needs less number of treatment compared to Q‐switched Nd:YAG laser [14, 15]. Furthermore, several studies have confirmed that 1064 nm QSNYL enhances skin barrier function [16, 17]. In our study, we found that with the combination of Q‐switch ruby laser and intradermal PRP injection, the pigmentation was regression in intensity and extension, and had a long‐term effective treatment measured by the improvement of VAS and Sadick tear trough rating scale. This result is consistent with a prior study, in which combined PRP injection with Er:YAG laser reduced melanin content and improved skin lightness and wrinkles compared to Er:YAG laser only [10].

We ordered an additional 24 weeks follow‐up observation to look for the recurrence and long‐time efficacy, and the result showed that the combined group had a better achievement. Such promising efficacy may attribute to the growth factors and bioactive molecules that PRP contains. Platelets play a critical role in homeostasis and wound healing by releasing of growth factors from alpha granules. They are storage pools of several growth factors such as platelet‐derived growth factor (PDGF), transforming growth factor beta (TGF‐β), vascular endothelial growth factor (VEGF), insulin‐like growth factor (IGF), fibroblast growth factor (FGF) and many cytokines, these growth factors can promote neoangiogenesis [12, 18]. In recent years, it has been confirmed that PRP can delay the activation of extracellular signal‐regulated kinase by TGF‐β1 and inhibit the expression of prostaglandin E2 and tyrosinase activity through EGF to improve the melanin deposition [19]. Also, laser therapy can be one of the reasons that can aggravate PIH, especially in the periorbital skin [20]. We hypothesized that intradermal injection of PRP after QSR laser therapy would lessen the risk of PIH base on its pathogenesis. The inflammatory response in epidermal can release prostaglandins and leukotrienes, resulting in stimulating melanocytes and increasing in the synthesis of melanin and transfer to surrounding keratinocytes [12]. The newly formed vasculars increased regional blood circulation and meet the demands for epidermal metabolism and lower inflammatory cytokine concentrations. We also found improvement of wrinkles, tear troughs, and local inflammation after PRP injection, these findings are in line with some other studies in PRP and skin rejuvenation [21, 22]. We believe that PRP injection combined with laser therapy is a highly effective treatment that deserves widespread use in clinical practice.

The major limitations of this study is the patient's sleeping status, patients who have a regular schedule may improve dark circles. And due to the occupational reasons, patients may stay more time indoors and exposed to UV light lesser, which may cause less post‐inflammatory hyperpigmentation.

No serious adverse events occurred. The main adverse effects reported were injection pain and erythema. These effects all resolved quickly after ice compression.

## 5 Conclusions

Combining with PRP injection can improve the therapeutic effect of Q‐switched ruby laser in treating periorbital hyperpigmentation and lessen the risk of post‐inflammatory hyperpigmentation, indicating a new option for POH treatment.

## Ethics Statement

The authors confirm that the ethical policies of the journal, as noted on the journal's author guidelines page, have been adhered to. No ethical approval was required as this is a review article with no original research data.

## Consent

Patients give permission to take photographs and/or videos and full rights to use the images resulting from the photography/video filming, and any reproductions or adaptations of the images for publicity or other purposes to help achieve the research aims.

## Conflicts of Interest

The authors declare no conflicts of interest.

## Data Availability

The data that support the findings of this study are available from the corresponding author upon reasonable request.
